# Evaluation of Febrile Seizures: A Therapeutic Review of Current Modalities

**DOI:** 10.7759/cureus.50947

**Published:** 2023-12-22

**Authors:** Brinda Patel, Mokshit M Shah, Amrita Suresh, Parag N Patel, Priyansh Patel, Siddharth Kamal Gandhi

**Affiliations:** 1 Department of Internal Medicine, Gujarat Medical Education and Research Society, Ahmedabad, IND; 2 Department of Internal Medicine, Gujarat Medical Education and Research Society, Patan, IND; 3 Department of Pediatrics, Kasturba Medical College, Mangalore, IND; 4 Department of Internal Medicine, Medical College Baroda, Vadodara, IND; 5 Department of Internal Medicine, M.P. Shah Government Medical College, Jamnagar, IND

**Keywords:** levetiracetam, midazolam, phenobarbitone, diazepam, simple febrile seizure, complex febrile seizures

## Abstract

As benign as its nature is, a febrile seizure (FS) can be one of the most frightening experiences for parents to witness. It is a seizure that occurs in infants and children aged six months to five years, accompanied by a fever (with a temperature of at least 100.4°F or 38.0°C by any method), without any infection in the central nervous system. FS is typically benign and tends to resolve on its own. Overall, the risk of recurrence after an FS is high, so there is still a sizable knowledge discrepancy that needs to be addressed for better understanding and management of the disease. Thus, the objective of this review is to evaluate current therapeutic modalities available for FS and summarize recent recommendations on the management of this condition. On June 25, 2023, a review was undertaken using the Medical Subject Headings Tool (MeSH), and the following keywords yielded 867 results: seizures, febrile/drug therapy [Mesh] and seizures, and febrile/therapy [Mesh]. A total of 21 relevant articles were chosen for the research. Seizures were classified as simple and complex FS (CFS) based on clinical features. CFS usually results in recurrence. Certain investigations like computed tomography (CT) scans, magnetic resonance imaging (MRIs), and electroencephalography (EEG) are helpful, along with laboratory investigations, to rule out other causes of FS. After reviewing the current literature, we have tried to conclude whether the current pharmacotherapy is effective in treating FS.

## Introduction and background

The incidence of febrile seizure (FS), which is the most prevalent form of seizure among children, is higher in developing countries [1]. The incidence of these conditions ranges from 2% to 5% among children, and they can be linked to a wide variety of infectious causes [2]. Fever-associated seizures are a major reason for pediatric outpatient clinic and emergency room visits, often resulting in significant distress for parents. It has been characterized as a seizure with fever (at least 100.4°F or 38.0°C with any method) in infants and children aged between six months and five years, without evidence of central nervous system (CNS) infection [3]. The most frequently observed type of seizure is the simple FS, which is characterized by generalized seizures that last for less than 15 minutes and do not recur within 24 hours without any prior neurological issues. If the FS episode is focused, prolonged, or multiple within the first 24 hours, it is considered a complex FS (CFS) [4]. Overall, the likelihood of experiencing a recurrence after FS is 32%. The majority of those who experience a recurrence will have only one additional episode (54%), while 28% will have two recurrences, and 18% will have three or more recurrences. Among those who experience recurrent seizures, 75% will do so within the first year after the procedure [5]. Regarding existing treatment methods and their efficacy, there is still a sizable knowledge discrepancy that needs to be addressed for better understanding and management of the disease. There’s also a great need to provide adequate parental education about the nature of the disease. Thus, the objective of this literature review is to evaluate current therapeutic modalities available for FS and summarize recent recommendations on the management of this condition to avoid frequent improper prescription of diagnostic tests and misuse of medicines in children with FS.

Methodology

A literature review searching databases like PubMed, Medline, PubMed Central, and Google Scholar was undertaken using keywords like seizures, Febrile seizures, therapy AND seizures, and Medical Subject Headings Tool (MeSH) yielded 867 results using the following keywords: “seizures, febrile/drug therapy” [Mesh] and “seizures, febrile/therapy” [Mesh]. Any duplicate publications and research unrelated to the human species were removed. Publications that were not written in English were also eliminated. The publications with full free-text reviews were chosen after the results were reviewed for text availability, titles, and abstracts. This reduced the number of articles to 126. Following that, each author analyzed the publications, and a total of 21 articles were chosen for the research.

## Review

Clinical features

FS typically occurs when a child’s temperature exceeds 38°C, though seizures can occur at any point during a febrile illness and may only develop after the child has experienced a fever. Common indicators of FS include a loss of consciousness, difficulty breathing, paleness or blueness of the skin, frothing at the mouth, eyes rolling back, a fixed stare, generalized or focal muscle spasms, and jerky movements of the arms and legs. Following a seizure, children may exhibit irritability, confusion, or drowsiness, but they typically recover within approximately 30 minutes [6-9]. There are two main forms of FS: simple FS, which constitutes 70% of all cases and generally does not lead to long-term neuro-developmental consequences, and CFS. The characteristics of simple and CFS are described in Table 1 [10].

**Table 1:** Characteristics of FSs FS, febrile seizure The information in this table has been adapted from Laino et al. [10].

Simple	Complex
Generalized tonic-clonic seizures without focal features	There are focal features in which, for example, only one side of the body is involved
Seizures last less than 10 minutes	Seizures last for more than 10 minutes
Seizures spontaneously resolve	Two or more seizures occur within 24 hours
There is no recurrence within 24 hours	Full recovery is not observed after one hour
-	There are postictal neurological consequences
-	There is a short period of paralysis, defined as Todd’s paralysis, after the seizure
-	FS develops
-	Anticonvulsant drugs may be required to interrupt the seizure

Investigations

To effectively assess and diagnose a child with FS who has experienced convulsions, it is crucial to carry out a comprehensive and precise history and clinical evaluation, incorporating a neurologic examination, to determine any secondary causes in the emergency department (ED) [11]. It is crucial to gather information about a child’s history from their parents or caregivers, including specifics about the nature, duration, and post-seizure phase of convulsions; recent infectious diseases or fevers; any use of antibiotic therapy; associated symptoms; immunization and vaccination records; a history of previous febrile seizures (FSs) or epilepsy diagnosis; any neurological conditions or diseases; family history of FSs, epilepsy, or neurological diseases; use of antipyretics; and the necessity of rescue anticonvulsants such as diazepam or midazolam [11]. Documenting vital signs, such as temperature, heart rate, respiratory rate, capillary refill time, and blood glucose, is crucial once stability has been ensured [8,9]. It is essential to differentiate between a first FS and the initial occurrence of either afebrile or epileptic convulsions. It is important to establish a clear history of fever, whether it occurred before or after the FS [12]. For any child exhibiting seizures, fever, and meningeal symptoms (including neck stiffness and Kernig and/or Brudzinski signs) or for those with a history or examination suggesting meningitis or intracranial infection, magnetic resonance imaging (MRI) followed by a lumbar puncture should be done [4]. This process should be specially performed on children displaying signs and symptoms of a severe ailment or intracranial infection, including pneumonia or meningitis/encephalitis. However, children without the signs and symptoms may show FS. Also, it is not mandatory for children aged one or above who have a recognized source of infection, have received all necessary vaccinations, and are experiencing a straightforward FS [13]. The outcomes of a research project indicated that children who experienced FSs had noticeably higher levels of neutrophils than those who had fever without seizures. Furthermore, children with FS exhibited lower levels of lymphocytes compared to those without seizures [14]. A retrospective study demonstrated the utility of multiplex polymerase chain reaction (PCR) analysis in identifying various viruses that can lead to FS in children. This approach has the potential to facilitate the risk stratification of these patients in the future, thereby reducing the unnecessary use of antibiotics [15]. For children who have experienced complex or recurrent FSs or display neurological abnormalities, doctors may use computed tomography (CT), MRI, electroencephalography (EEG), or a combination of these techniques to determine if there are any underlying neurological conditions. However, if a healthy child has a clear source of infection, an EEG should not be performed after an FS. If an EEG is administered, it should be done no sooner than 48 hours post-FS to prevent misinterpreting post-ictal electrical activity as abnormal electrical activity [4]. A retrospective study conducted by Harini et al. found that an epileptiform EEG was not a reliable predictor of epilepsy in neurologically healthy or mildly delayed children with their first CFS, as it had a low positive predictive value [16].

Treatment

As benign as it is, treating the FS occurrence is as important as treating its recurrence, as the risk of recurrence of FS is almost 32% in patients who have suffered at least one attack of FS [10]. Reviews of different treatment plans have been done, and various articles have been published on the basis of different studies that were performed on children aged six months to 60 months [10]. While uncomplicated FS generally have a benign course and prognosis, there is a consensus that prompt treatment is necessary [10]. A review in the International Journal of Environmental Research and Public Health identifies red flags for further management of children presenting with FS [10]. These include children with CFS, a positive Kernig’s sign and or positive Brudzinski sign, neck stiffness, altered consciousness for more than one hour, non-blanching rashes in children with fever, bulging fontanelle, tachycardia, and signs of respiratory distress (including low oxygen saturation, tachypnea, grunting, and chest wall recessions) [10].

Review of different treatment modalities

In a prospective randomized controlled trial (RCT), it was determined that both diazepam and midazolam are equally safe and effective for managing FS [10]. The study revealed that the mean time from hospital arrival to the initiation of treatment was significantly shorter in the midazolam group compared to the diazepam group [10]. Additionally, the average time to achieve seizure control was significantly shorter in the midazolam group compared to the diazepam group. Notably, no significant side effects were observed in either treatment group [10].

In another RCT held in China on the reduction of febrile recurrence by intermittent oral levetiracetam, a significant difference was observed between the two groups. During the study of 48 weeks, one patient in the levetiracetam group exhibited severe drowsiness. No other side effects were observed in the same patient and other children [17]. Given that intermittent oral levetiracetam was deemed effective in preventing FS recurrence and was safe for children with reduced wastage of medical resources, it was proposed to use intermittent oral levetiracetam as preventive therapy [17].

The carbon dioxide (CO2) against febrile (CARDIF) seizures trial is a mono-centric, prospective, double-blind, placebo-controlled RCT, which was conducted to examine the evidence of superiority of the carbogen inhalation through low-pressure flask with a mask containing 6 L carbogen (5% CO2 plus 95% oxygen) compared to placebo inhalation, to suppress an FS recurrence within three minutes [18]. In this study, as a result, almost 80% of children who had an episode of FS did not experience any type of recurrence of the seizure. To our knowledge, CARDIF was the only registered clinical trial that investigated a new treatment to suppress an acute recurrence of an FS till 2013 [18].

According to a prospective study conducted in Israel from January 2008 to March 2010, it was concluded that intravenous (IV) diazepam or midazolam is more beneficial than diazepam suppository or rectal diazepam. Also, phenobarbital was not recommended during this study as it was not found useful [19].

Another RCT performed from December 2004 through March 2006 concluded a significant difference in the rate of recurrence of FS between children treated with a diazepam suppository and those who were not. As a conclusion of this study, we can say that the diazepam suppository after an FS will reduce the incidence of recurrent FS during the same febrile illness [20].

A single-center retrospective study conducted in Japan in 2020, among six- to 60-month-old children, suggested that when the use of rectal diazepam was decreased, there was a significant rise in hospital revisits for the cases of recurrences of FS [21]. In another RCT study done in the Netherlands on the prophylactic use of ibuprofen syrup for a reduction in the recurrent attack of FS, no significant reduction in the rates of recurrence of FS was noticed by giving ibuprofen syrup prophylactically with the increasing body temperature in children [22].

**Figure 1:**
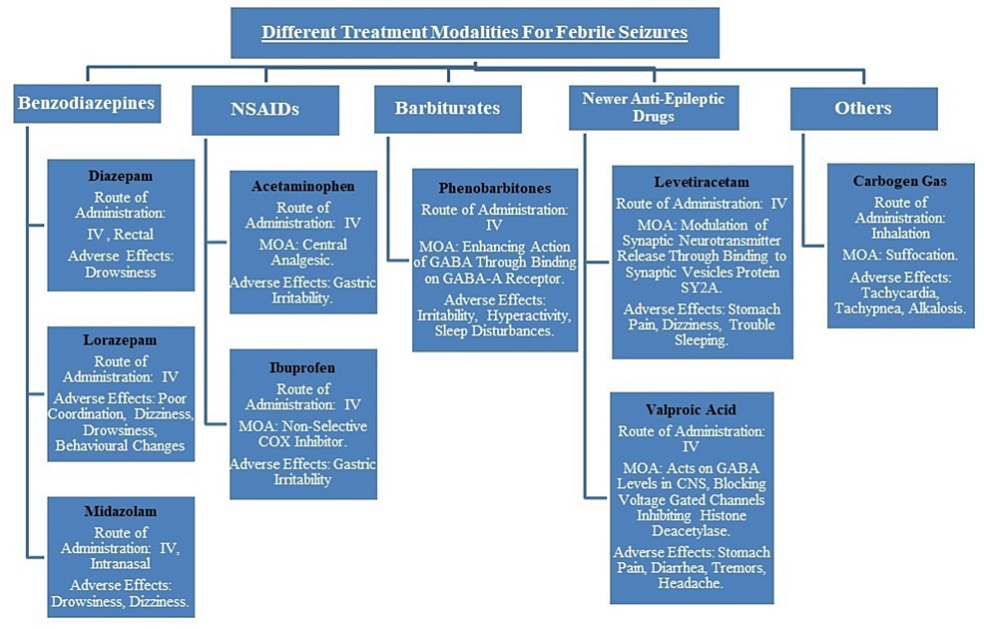
Therapeutic modalities for febrile seizures NSAIDs, non-steroidal anti-inflammatory drugs; IV, intravenous; MOA, mechanism of action; COX, cyclooxygenase; GABA, gamma amino butyric acid; CNS, central nervous system

A study was performed in Israel where the efficacy of intranasal midazolam was compared with IV diazepam. In this study, as a conclusion, both the drugs were found equally effective in children; however, seizures were controlled more quickly with IV diazepam than with intranasal midazolam, although midazolam was as safe and effective as diazepam [23]. While IV diazepam is effective, it can be challenging to administer to children experiencing convulsions. To explore an alternative approach, the rectal administration of diazepam was employed [24]. A study conducted in Denmark revealed that rectal administration of diazepam was successful in 80% of cases, with diazepam failing in 10% of cases. In the remaining 10%, the convulsions were resistant to all forms of diazepam administration [24]. The review of different treatment modalities is shown in Figure 1.

Prevention and prognosis

Recurrence rate has multiple risk factors that include age at onset, family history of epilepsy, or febrile convulsions [24]. Short-term therapy with diazepam for 18 months was found to be useful in the prevention of FS. However, children who were left untreated were grouped into three categories based on the number of risk factors present. The recurrence rate was 80-100%, if risk factors were more than three; 50%, if two risk factors were present; 25%, for children with one risk factor; and 12%, for children with no risk factors. The study suggested the use of diazepam as a prophylactic agent at times of fever based on these three categories [24]. According to a study done in Japan from 2011 to 2018, there was an increase in the recurrence of FS with a decrease in the dose of diazepam compared to the year. This concludes the potential use of rectal administration of diazepam as a prophylactic agent [21]. Another comparative double-blind trial was done to analyze the difference between the continuous use of sodium valproate and phenobarbitone in the prevention of simple FS. The statistical analysis revealed a significant difference between the sodium valproate group and the untreated group, as well as between the phenobarbitone group and the untreated group. However, the difference between the valproate and phenobarbitone groups was found to be statistically insignificant. The children, however, had multiple side effects from both drugs [25]. A review done by Offringa et al. concluded zinc to be of no use in the prevention of FS. They also concluded that prompt treatment of every febrile episode with an antipyretic or antiepileptic drug was not useful in the prevention of FS [26].

## Conclusions

In this review article, we assess the various therapeutic methods currently available for treating FS. We delved into the clinical features of both simple FS and CFS. Furthermore, discussion led to numerous investigations done on a regular basis to establish cause and their rationale in FS. Based on the studies reviewed, benzodiazepines administered via IV, rectal, and intranasal routes form the mainstay of medical treatment. For prophylactic management and prevention of further recurrence, chronic administration of drugs like levetiracetam, valproic acid, and carbogen gas have also played some role in reducing the intensity and recurrence of seizures, while antipyretic drugs like ibuprofen and paracetamol showed no help in reducing the temperature or recurrence of an episode of FS. So far, the value of multiplex PCR analysis in the identification of a number of viruses that can cause FS in children and its potential to avoid the misuse of antibiotics are still in the early phases. Furthermore, CARDIF was the only registered clinical trial that investigated a new treatment modality, carbogen, to suppress an acute recurrence of FS. Thus, FS is a challenging disease to treat, and patients often need a multifaceted approach. The evidence for most of these treatments is modest, and future RCTs and additional longitudinal data are needed to establish the most efficient interventions for FS management.
